# Withaferin A Associated Differential Regulation of Inflammatory Cytokines

**DOI:** 10.3389/fimmu.2018.00195

**Published:** 2018-02-09

**Authors:** Seema Dubey, Hyunho Yoon, Mark Steven Cohen, Prakash Nagarkatti, Mitzi Nagarkatti, Dev Karan

**Affiliations:** ^1^Department of Pathology, Microbiology and Immunology, University of South Carolina School of Medicine, Columbia, SC, United States; ^2^Department of Surgery, University of Michigan, Ann Arbor, MI, United States

**Keywords:** inflammation, inflammasome, withaferin A, cytokines/chemokines, macrophages

## Abstract

A role of inflammation-associated cytokines/chemokines has been implicated in a wide variety of human diseases. Here, we investigated the regulation of inflammatory cytokines released by monocyte-derived THP-1 cells following treatment with the dietary agent withaferin A (WFA). Membrane-based cytokine array profiling of the culture supernatant from adenosine triphosphate-stimulated WFA-treated THP-1 cells showed differential regulation of multiple cytokines/chemokines. A selected group of cytokines/chemokines [interleukin-1 beta (IL-1β), CCL2/MCP-1, granulocyte-macrophage colony stimulating factor, PDGF-AA, PTX3, cystatin-3, relaxin-2, TNFRSF8/CD30, and ACRP30] was validated at the transcription level using qPCR. *In silico* analysis for transcriptional binding factors revealed the presence of nuclear factor-kappa B (NF-κB) in a group of downregulated cytokine gene promoters. WFA treatment of THP-1 cells blocks the nuclear translocation of NF-kB and corresponds with the reduced levels of cytokine secretion. To further understand the differential expression of cytokines/chemokines, we showed that WFA alters the nigericin-induced co-localization of NLRP3 and ASC proteins, thereby inhibiting caspase-1 activation, which is responsible for the cleavage and maturation of pro-inflammatory cytokines IL-1β and IL-18. These data suggest that dietary agent WFA concurrently targets NF-κB and the inflammasome complex, leading to inhibition of IL-1β and IL-18, respectively, in addition to differential expression of multiple cytokines/chemokines. Taken together, these results provide a rationale for using WFA to further explore the anti-inflammatory mechanism of cytokines/chemokines associated with inflammatory diseases.

## Introduction

Events of inflammation greatly impact the pathogenesis of various disease types including cancer. Cytokines and chemokines actively mediate immune cell interactions, leading to the initiation of inflammatory responses. Macrophages, a type of immune cell, produce a number of cytokines and chemokines in response to infection or severe chronic conditions (1, 2). The dual role of macrophages in both anti- and pro-inflammatory processes is well documented (3). Pro-inflammatory activation of macrophages is involved in the regulation of various inflammatory disorders including autoimmune diseases (4, 5). Therefore, macrophage-associated cytokines/chemokines are considered preventive and therapeutic targets in inflammatory diseases, and they are being tested in clinical trials (6).

Secretion of inflammatory cytokines/chemokines is controlled by diverse regulators such as transcription factors, toll-like receptors, and recently inflammasomes. Among transcription factors, nuclear factor-kappa B (NF-κB) is the most studied regulator for the modulation of inflammatory cytokines in various cell types. Constitutive activation of NF-κB has been detected to increase NF-κB-related gene expression including inflammatory cytokines/chemokines such as CCL20/MIP-3α and granulocyte-macrophage colony stimulating factor (GM-CSF) (7). Although the mechanistic regulation of inflammatory events involves a complex network of molecules, recent studies showed that inflammasomes are considered master regulators of inflammation-associated signaling. The inflammasome is a multiprotein complex consisting of NOD-like receptor (NLR), the adaptor protein ASC, and procaspase-1. Upon stimulation, inflammasome assembly leads to activation of caspase-1, which is responsible for the cleavage of mature and secretory forms of interleukin-1 beta (IL-1β) and IL-18, inducing a variety of biological effects associated with inflammation and other disease processes (8–10).

To understand the mechanistic regulation of inflammation-associated cytokines/chemokines, we investigated the impact of the dietary agent withaferin A (WFA) using the human monocytic cell line THP-1. WFA, a steroidal lactone derived from *Withania somnifera*, has been shown to display multiple activities including anti-inflammatory, antiproliferative, proapoptotic, and antitumorigenic (11–14). It has been reported that WFA can modulate various target molecules by direct or indirect interactions, and several such targets include NF-κB, protein kinase C, JNK, AKT, and ERK (15–20). In this study, we showed that WFA differentially regulates multiple cytokines/chemokines in lipopolysaccharide (LPS)-primed adenosine triphosphate (ATP)-stimulated THP-1 cells. We further demonstrated a dual mechanism of inflammatory cytokine regulation *via* NF-κB inhibition and the redistribution of inflammasome complex proteins.

## Materials and Methods

### Cell Line and Reagents

The human monocyte THP-1 cell line (TIB-202) was purchased from ATCC (Manassas, VA, USA) and was maintained in Dulbecco’s Modified Eagle Medium supplemented with 10% fetal bovine serum, 1% glutamine, 1% sodium pyruvate (Invitrogen), and 50 µg/ml gentamicin (Mediatech) in a tissue culture incubator at 37°C with 5% CO_2_ and 95% relative humidity. WFA was purchased from ChromaDex. Adenosine triphosphate (ATP), Nigericin, monosodium urate (MSU), and phorbol myristate acetate (PMA) were from Sigma-Aldrich; LPS was purchased from eBioscience. Anti-NLRP3 and ASC antibodies were purchased from Millipore and AdipoGen, respectively. Antibodies to NF-κB, p65 and Histone H3 were obtained from Abcam; antibodies to IL-1β, IL-18, caspase-1, β-actin, and secondary antibodies were purchased from Santa Cruz Biotechnology.

### Human Cytokine Arrays and Image Analysis

Human cytokine profiling was performed using a Proteome Profiler Human XL Cytokine Array Kit (R&D Systems), which detects 102 human soluble cytokines. THP-1 cells were seeded at a density of 5 × 10^5^ cells per well in a six-well plate in the presence of PMA (500 ng/mL) to differentiate the cells into macrophages (21, 22). Following overnight incubation and addition of LPS (200 ng/mL) for 2 h, ATP (1 mM) was added to the cultured cells to induce cytokine production in the presence or absence of WFA (20 µM). Following incubation for 4 h, culture supernatant was collected, and the cells were lysed to isolate RNA. Human XL Cytokine Arrays were incubated overnight at 4°C with 1.5 mL of the cell culture supernatant, and the procedure was performed according to the manufacturer’s instructions. Following incubation with a detection antibody cocktail, antibody conjugation, and recommended washes, the immunoblots on the membrane were developed with Chemiluminescent Substrate Reagent Kit (Invitrogen) and were exposed to X-ray film. Cytokine array TIF file images were analyzed using ImaGene 8.0 software (BioDiscovery, Inc.). After uploading images into the software, a grid file was manually over imposed on them to assign spot gene identities. In the next step, spot finding and automated segmentation-proprietary algorithms were used to determine both signal and background intensities. For background correction, median background intensities were subtracted from mean signal intensities. To further calculate the differential expression, the mean signal intensity of the cytokine/chemokine spots was divided by the mean signal intensity of the reference spots (*n* = 6) for each membrane.

### Screening for NF-κB Transcriptional Factor Binding Sites

**Figure 1:**
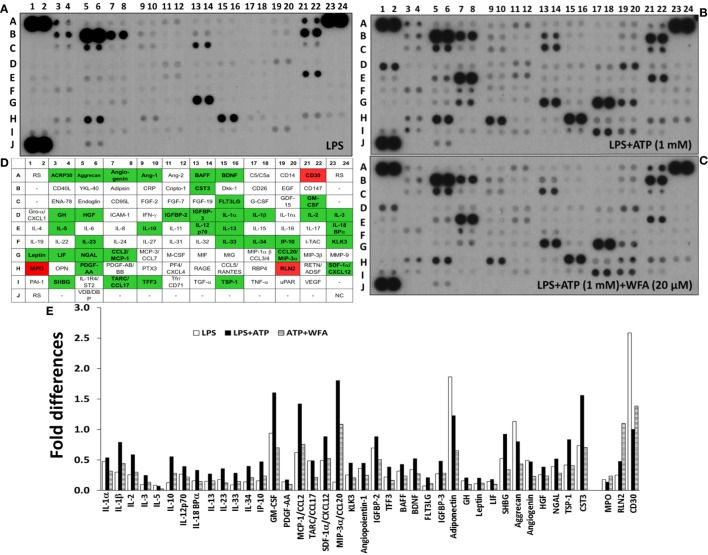
Differential cytokine/chemokine production levels modulated *via* withaferin A (WFA) in THP-1 cells. THP-1 cells at a density of 5 × 10^5^ cells/well were seeded in six-well plates in the presence of phorbol myristate acetate (500 ng/mL). Following overnight incubation, lipopolysaccharide (LPS) (200 ng/mL) was added for 2 h, and the cells were primed with adenosine triphosphate (ATP) (1 mM) for 30 min. Collective analysis of 102 cytokines in the culture supernatant from THP-1 cells treated with LPS [**(A)** control] primed with ATP **(B)** and treated with WFA **(C)**. **(D)** A schematic representation of the cytokine/chemokine spot positions in duplicate on the membrane with respective internal controls. Rectangles filled with green background represents downregulation (>1.5-fold), and with red background represents upregulation (>1.5-fold) of cytokines/chemokines following WFA treatment. RS, reference spots; NC, negative controls; **-**, blank (no spot). **(E)** The relative fold change in differentially expressed genes (>1.5-fold). The quantitative analysis of the pixel intensity data did not provide *P*-values to demonstrate significance levels.

Based on Figure 1E, the gene coding promoter regions for some of the cytokines/chemokines, IL-1β, GM-CSF, B-cell activating factor (BAFF), sex hormone binding globulin (SHBG), chemokine (C-C motif) ligand (CCL2/MCP-1, and CCL17/TARC), cystatin-3 (CST3), thrombospondin-1 (TSP-1), relaxin-2 (RLN2), tumor necrosis factor receptor superfamily 8 (TNFRSF8/CD30), and adiponectin (ACRP30), were screened for NF-κB transcription factor binding sites using MatInspector software 8.4 with Matrix Library 10.0 from the Genomatix software suite (http://www.genomatix.de/cgi-bin/eldorado/main.pl). The parameters for searching the database for NF-κB binding sites were set at core similarity of 1.00 (maximum) with optimized matrix similarity.

### RNA Isolation and Real-time PCR

Total RNA was isolated from the THP-1 cells using an RNA isolation kit (RNeasy Mini Kit, Qiagen), and cDNA was synthesized by reverse transcriptase (Superscript II; Invitrogen). The level of mRNA expression of the differentially expressed cytokines was performed using FAST SYBR Master Mix on StepOnePlus Real-Time PCR (Applied Biosystems). The primer sequences to amplify the cytokine gene products are given in the Table S1 in Supplementary Material.

### Preparation of Cell Lysates for Protein Expression Analysis

Cell lysates were prepared with RIPA buffer (Sigma-Aldrich) using the standard protocol. Nuclear and cytoplasmic fraction analysis from the THP-1 cells was performed with nuclear and cytoplasmic extraction reagents (Thermo Scientific) according to the manufacturer’s protocol. Briefly, the harvested cells were suspended in lysis buffer containing a proteinase inhibitor cocktail (Sigma-Aldrich) and centrifuged at 16,000 × *g* for 5 m. The supernatant was saved for cytoplasmic extracts, and the pellet was re-suspended in a special buffer (NER) for the nuclear fraction. After being centrifuged at 16,000 × *g* for 10 m, the supernatant was collected for nuclear extract. The collected supernatant from the total cell lysates or the cytosol and nucleus were subjected to SDS-PAGE western blot analysis and transferred to a polyvinylidene difluoride membrane (Bio-Rad). The membranes were incubated with the indicated primary antibodies at a dilution of 1:500 and HRP-conjugated secondary antibodies (dilution 1:2,000). The immune-reactive bands were visualized using a chemiluminescent substrate (Invitrogen) and were exposed to X-ray film.

### Enzyme Linked Immunosorbent Assay (ELISA)

Culture supernatant was harvested from the THP-1 cells following treatments, and ELISA assays for human caspase-1, IL-1β (R&D Systems), and IL-18 (MBL) were performed using ELISA kits following manufacturer’s instructions.

### Analysis of NLRP3 Inflammasome Complex Protein Localization

THP-1 cells were seeded with 50% confluence on a coverslip in six-well plates. While in the wells, the cells were treated with nigericin with or without WFA. After removing the medium, cells were washed with phosphate-buffered saline (PBS) and then fixed with 2% paraformaldehyde for 10 min. Following subsequent washing and blocking with 5% bovine serum albumin (BSA) in PBS, the cells were incubated with ASC or NLRP3 antibody in 1% BSA for 60 min. After rinsing with 1% BSA in PBS, the cells were incubated with secondary antibody (1:500) Alexa Fluor 488 (green-fluorescent dye from Life Technologies) and Cyanine Cy^TM^3 (red-fluorescent dye from Immune Research) for 60 min. Cells were rinsed with 1% BSA in PBS, and then incubated with DAPI for nuclear staining for 5 min at room temperature. The cells were rinsed, and mounted using fluorescence mounting medium (Dako). The slides were analyzed under the confocal scanning laser microscope (Zeiss LSM 510 Meta).

### Statistical Analysis

We used the “*t*-test” of unequal variance, and one-way analysis of variance (ANOVA) to calculate the differences among treatments. For the ANOVA, the comparisons between each treatment was analyzed using LPS treatment as control, and corrected for multiple comparisons by applying Dunnett’s test. All the statistical analyses were performed using GraphPad Prism 6 software.

## Results

### Effect of WFA on Inflammatory Cytokine Production in THP-1 Cells

We used a membrane-based sandwich immunoassay (Proteome Profiler Human XL Cytokine Array) to examine the expression profile of inflammatory cytokines. Culture supernatant from the human THP-1 cell derived macrophages treated with or without WFA was hybridized on cytokine array membranes. The cytokines/chemokines spot intensity signals are shown in Figure 1 (Figure 1A: LPS control, Figure 1B: ATP primed cells, and Figure 1C: WFA treatment). Adenosine triphosphate (ATP), an activator of the NLRP3 inflammasome, was used for promoting cytokine secretion by the THP-1 cells. Following image analysis and the quantification of pixel intensity for each spot, 41 cytokine genes showed differential expression of >1.5-fold between THP-1 cells treated with or without WFA (Figures 1B,C). The names of these differentially expressed 41 cytokines/chemokines are presented on a schematic grid that corresponds to the spot positions on the membrane (Figure 1D). Of these 41 cytokines, 38 (IL-1α, IL-1β, IL-2, IL-3, IL-5, IL-10, IL-12p70, IL-18BPα, IL-13, IL-23, IL-33, IL-34, IP-10, GM-CSF, PDFG-AA, CCL2/MCP-1, CCL17/TARC, SDF1α/CXCL12, CCL20/MIP-3α, KLK3, angiopoientin-1, IGFBP-2, TFF3, BAFF, BDNF, FLT3LG, IGFBP-3, ACRP30/adiponectin, GH, Leptin, LIF, SHBG, aggrecan, angiogenin, HGF, NGAL, TSP-1, and CST3) were downregulated, and 3 (MPO, RLN2, and TNFRSF8/CD30) were upregulated in ATP-stimulated WFA treated THP-1 cells (Figure 1E).

### *In Silico* Analysis of NF-κB Binding Sites in Cytokines/Chemokines

To understand the differential expression of these cytokines and to identify a common target, we screened the gene coding promoters of the randomly selected 11 cytokines/chemokines. Transcriptional analysis showed that 6 (IL-1β, GM-CSF, CCL2/MCP-1, BAFF, SHBG, and ACRP30/adiponectin) of the 9 downregulated cytokines contain NF-κB binding sites (Table 1). Three cytokines (CST3, CCL17/TARC, and TSP-1) lack NF-κB binding sites on their promoter region; however, analysis of CCL17/TARC and TSP-1 showed the c-Rel transcriptional factor, which co-cites with NF-κB. In contrast, two upregulated cytokine genes (RLN2 and TNFRSF8/CD30) neither co-cite with nor contain NF-κB sites. This analysis leads to the hypothesis that a collective decrease in production of a group of cytokines/chemokines may be regulated *via* a common factor, NF-κB.

**Table 1:** Analysis of nuclear factor-kappa B (NF-κB) transcriptional binding sites on the differentially expressed cytokine genes following adenosine triphosphate stimulated and treatment with withaferin A of THP-1 cells.

		Position						
Name	Gene ID	From	To	Anchor	Strand	Core similarity	Matrix	Sequence
Interleukin-1 beta	3553	827	841	834	(+)	1.0	0.891	gtGGGAaaatccagt
		827	841	834	(−)	1.0	0.974	actggattTTCCcac

Granulocyte-macrophage colony stimulating factor	1437	291	305	298	(+)	1.0	0.832	gagGGGAgtcactca
		417	431	424	(−)	1.0	0.905	gaGGGAatgaccacc
		895	909	902	(+)	1.0	0.924	ccaggagaTTCCaca
		912	926	919	(−)	1.0	0.904	ggGGGAactacctga

CCL2/MCP-1	6347	389	403	396	(+)	1.0	0.880	tgGGGAatttacaga
		667	681	674	(+)	1.0	0.913	ctGGGAggccccttg
		923	937	930	(−)	1.0	0.879	ctgtggatTTCCagg
		981	995	988	(−)	1.0	0.928	gaGGGAtcttccatg
		1672	1686	1679	(−)	1.0	0.944	ctgGGGAgccccact

BAFF	10673	130	144	137	(+)	1.0	0.936	tgGGGAatgtccagg
Sex hormone binding globulin	6462	96	110	103	(−)	1.0	0.846	cgGGGAtttcaccac
		404	418	411	(−)	1.0	0.892	aaGGGAcctgccccc

Adiponectin/ACRP30	9370	743	757	750	(−)	1.0	0.939	tgGGGAagttcctgg
		743	757	750	(+)	1.0	0.911	ccaggaacTTCCcca

CCL17/TARC^a^	6361	No NF-κB binding site				1.0		

TSP-1^a^	7057	No NF-κB binding site				1.0		

Cystatin-3	1471	No NF-κB binding site				1.0		

Relaxin-2	6019	No NF-κB binding site				1.0		

TNFRSF8/CD30	943	No NF-κB binding site				1.0		

*^a^Represents genes co-cited with NF-κB; position refers to the predicted NF-κB transcriptional factor binding site within the promoter sequence of the respective gene; anchor refers the center position of the binding site; Strand is the input sequence on which the binding has been identified; Core similarity of maximum (1.0) represents highest conserved bases of a matrix match exactly in the sequence; Matrix similarity = 1: only if the candidate sequence corresponds to the most conserved nucleotide at each position of the matrix*.

### Validation of Differentially Expressed Cytokines following WFA Treatment of THP-1 Cells

**Figure 2:**
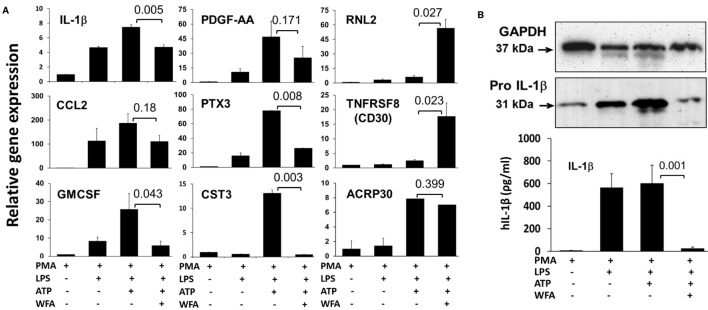
Representation of relative mRNA expression by quantitative real-time PCR **(A)**. Total RNA was isolated from THP-1 cells seeded as before with or without withaferin A (WFA) (20 µM) treatment and was subjected to real-time PCR to validate the differential expression of the identified cytokines. Data are presented as relative target gene expression with GAPDH normalization. **(B)** The validation of reduced interleukin-1 beta (IL-1β) by western blot on cell lysates (upper panel), and enzyme-linked immunosorbent assay in the culture supernatant (lower panel) from THP-1 cells primed with adenosine triphosphate and treated with WFA (20 µM). The experiment was repeated twice with similar results from independent cell preparations.

To validate the effect of WFA on the modulation of targeted cytokines/chemokines, we performed quantitative real-time PCR on samples harvested from THP-1 cells following treatment with or without WFA in presence of ATP. These data showed that the mRNA levels of RLN2 (*P* = 0.027) and TNFRSF8/CD30 (*P* = 0.023) in LPS-primed ATP-stimulated THP-1 cells were significantly induced by the WFA treatment, while IL-1β (*P* = 0.005), GM-CSF (*P* = 0.043), PTX3 (*P* = 0.008), and CST3 (*P* = 0.003) were significantly reduced compared to ATP-treated controls (Figure 2A). ATP-induced IL-1β downregulation due to WFA was further validated at the protein levels by ELISA assay (*P* = 0.001) and western blot (Figure 2B). Although non-significant, downregulation of CCL2/MCP-1 (*P* = 0.18) and PDGF-AA (*P* = 0.171) was also observed following WFA treatment. Thus, the mRNA expression levels corroborate the observations of differentially expressed cytokines/chemokines on the array as in Figure 1. The mRNA transcript level for ACRP30/adiponectin (*P* = 0.399) remained unchanged following WFA treatment, and it did not match the observed expression on the cytokine array. It is possible that WFA could regulate ACRP30/adiponectin expression at the protein level and may not affect the mRNA transcript; however, more studies are required to establish this observation.

### WFA Inhibits NF-κB Activation in THP-1 Cells

**Figure 3:**
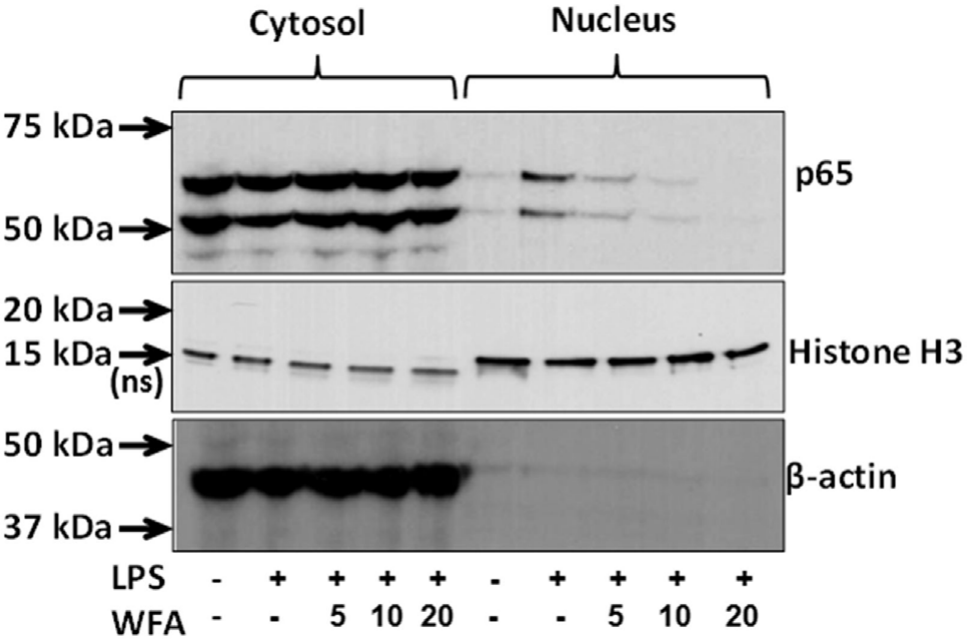
Inhibition of nuclear factor-kappa B (NF-κB) activation by withaferin A (WFA). Cell lysates from the cytoplasmic and nuclear fractions of lipopolysaccharide (LPS)-stimulated THP-1 cells following WFA (5, 10, 20 µM) treatment were subjected to protein expression analysis with antibodies for NF-κB, β-actin, and Histone H3 (a nuclear marker). NF-κB signaling molecules at the expected molecular weights of 50 and 65 kDa were observed both in both the cytosolic and nuclear fractions. The estimated size of 18 kDa for Histone H3 protein in nuclear lysate serves as internal control, whereas a non-specific band at ~15 kDa in the cytoplasmic fraction was also observed. Beta-actin (43 kDa) serves as an internal control for the cytoplasmic lysates. The experiment was performed twice with similar observations from two independent cell preparations.

*In silico* promoter analysis revealed that the downregulated cytokines/chemokines possess either NF-κB transcriptional binding sites or co-cites with NF-κB. Therefore, to gain an insight into collective downregulation of multiple cytokines, we examined the activity of NF-κB on nuclear and cytoplasmic fractions in LPS-primed THP-1 cells following WFA treatment. These data showed the inhibition of NF-κB translocation following WFA treatment, supporting that the collective downregulation of the identified cytokines/chemokines may be due to inhibition of NF-κB activation (Figure 3).

### Analysis of WFA’s Effect on NLRP3 Inflammasome Complex Proteins

**Figure 4:**
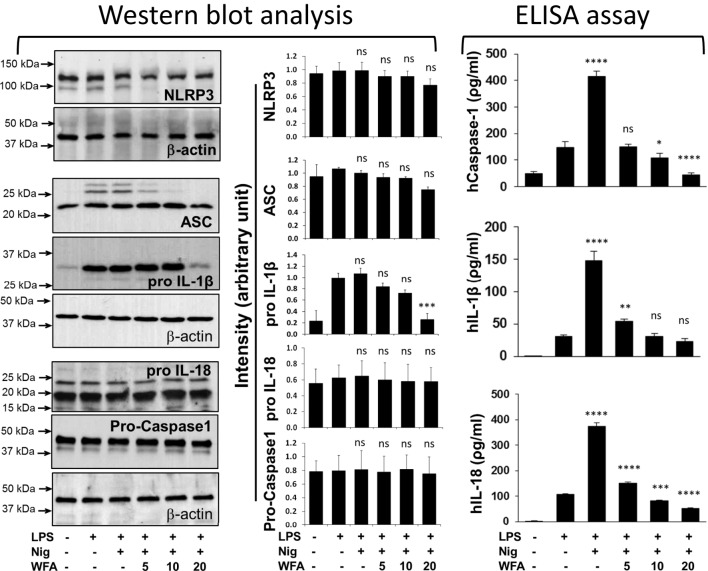
Effects of withaferin A (WFA) on proteins of the NLRP3 inflammasome complex and its downstream targets following nigericin stimulation of THP-1 cells. WFA inhibits the production of IL-18, interleukin-1 beta (IL-1β), and caspase-1, in a dose-dependent manner. Lipopolysaccharide (LPS)-primed THP-1 cells (5 × 10^5^ cells) were stimulated with nigericin (10 µM) for 30 min. Following incubation, different doses of WFA (5, 10, 20 µM) were added for 4 h. Cellular proteins were harvested and assayed for the protein levels of NLRP3, ASC, pro-caspase-1, IL-1β, and IL-18 by western blot analysis, while the production of active caspase-1, IL-1β, and IL-18 in culture supernatant was determined by enzyme-linked immunosorbent assay (ELISA). For protein analysis, the blots were hybridized multiple times with different target antibody. The density of each band of a specific protein (NLRP3, ASC, pro-caspase-1, pro IL-18, and pro IL-1β) was divided by the density of the β-actin band for that specific membrane. Statistical comparisons were performed with the relative density to determine statistical differences using one-way analysis of variance (ANOVA), and the significant levels are shown as compared with LPS treatment (control). The data represent mean ± SE from three independent experiments. ELISA results were also subjected to one-way ANOVA, and the significant levels are shown as compared with LPS treatment. *Significant level; ns, non-significant.

Using the NLRP3 inflammasome-specific stimulator nigericin, we sought to examine the effect of WFA on the NLRP3 inflammasome, which consists of three proteins (NLRP3, ASC, and pro-caspase-1). Interestingly, we did not observe any significant change in the protein expression levels of NLRP3 (*P* = 0.712), ASC (*P* = 0.319), or pro-caspase-1 (*P* = 0.999) (Figure 4). However, the downstream target protein pro-IL-1β was significantly reduced (*P* = 0.002) in a dose-dependent manner, while pro-IL-18 remained unchanged (*P* = 0.999). The analysis of variance (ANOVA) further revealed that the dispersion of the means across various treatments was minimal for NLRP3 (*F* = 0.585), ASC (*F* = 1.695), pro-caspase-1 (*F* = 0.012), and IL-18 (*F* = 0.033). Analysis of active and mature forms of caspase-1, IL-1β, and IL-18 revealed that WFA significantly inhibits the secretion of caspase-1 (*P* < 0.0001), IL-1β (*P* < 0.0001), and IL-18 (*P* < 0.0001) in THP-1 cells induced by nigericin (Figure 4). Based on the ANOVA, *F*-value indicates that the dispersion of the mean differences between the treatments was maximum for IL-18 (*F* = 1516.0) as compared to caspase-1 (*F* = 237.4) and IL-1β (*F* = 190.4). Similar results were obtained by using MSU, which is also an NLRP3 inflammasome-specific stimulator (Figure S1 in Supplementary Material).

**Figure 5:**
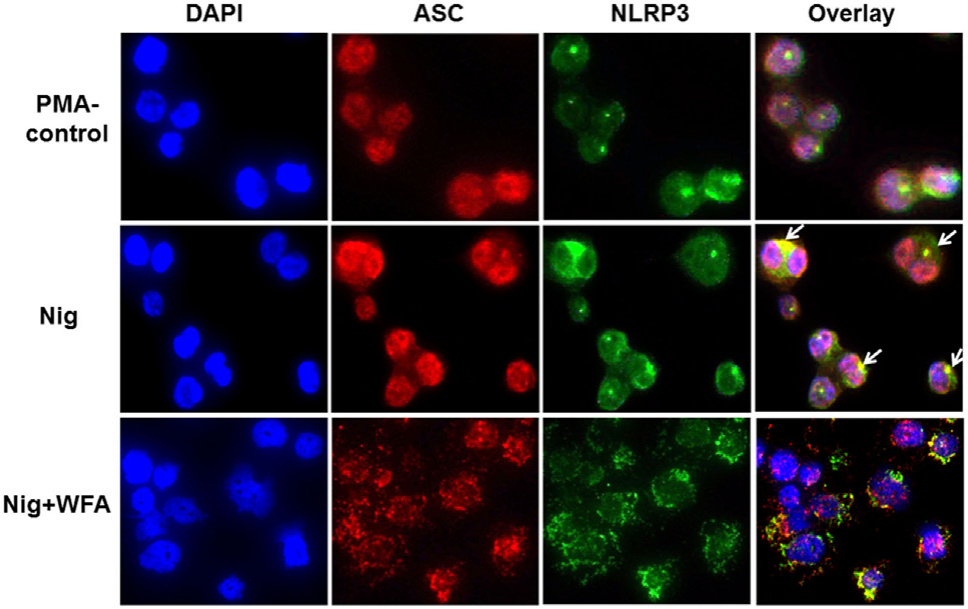
Representation of confocal fluorescence images (magnification 40×) of THP-1 cells analyzing the effect of dietary agent withaferin A (WFA) following NLRP3 inflammasome activation using nigercin. ASC and NLRP3 proteins were visualized by using Cyanine Cy^TM^3 (red-fluorescent) and Alexa Fluor 488 (green-fluorescent) dye labeled antibodies, respectively. DAPI was used for nuclear staining. A merged image of ASC and NLRP3 proteins shows co-localization of inflammasome complex proteins (yellow, marked by arrow). The experiment was performed four times from independent cell preparations.

Further examination of the localization of inflammasome complex proteins showed that NLRP3 and ASC are located both in the cytoplasm and the nucleus; however, the co-localization of ASC and NLRP3 seems outside the nucleus (Figure 5; PMA control). Following NLRP3 inflammasome activator nigericin, the co-localization of NLRP3 and ASC appeared more dominant outside around the nuclear membrane (Figure 5; Nig). This strong co-localization due to nigercin activation may be necessary for the inflammasome complex activity leading to increased secretion of caspase-1, IL-1β, and IL-18 (Figure 4). WFA treatment of the nigercin-primed THP-1 cells showed an altered distribution and disintegrated pattern of both co-localized and individual NLRP3 and ASC proteins inside and outside of the nucleus (Figure 5). Because of such unevenly distributed and fragmented localization of NLRP3 and ASC, it is likely that WFA blocks the activity of NLRP3 inflammasome complex, and hence diminishing the secretion of downstream targets (caspase-1, IL-1β, and IL-18). To further support our observations, we also analyzed the localization of NLRP3 and ASC following ATP (an activator of the NLRP3 inflammasome) stimulation of THP-1 cells with or without WFA. Similar results of altered and fragmented localization of NLRP3 and ASC proteins were observed following WFA treatment (Figure S2 in Supplementary Material).

## Discussion

Since a wide array of diseases, including cancer, are rooted in inflammation, there is a surge in interest in understanding the mechanistic regulation of inflammatory responses. In this study, we investigated the effect of the dietary agent WFA on differential regulation of inflammatory cytokines/chemokines using human monocytic THP-1 cells. Although various cell types produce inflammatory cytokines, macrophages express a number of cytokines in response to diverse stimuli including growth factors, survival, or danger signals (23, 24). We showed that WFA treatment modulates the level of multiple cytokines in LPS-primed ATP-activated THP-1 cells. We observed the effect of WFA upregulating the production of TNFRSF8/CD30, a tumor necrosis factor receptor shown to limit the proliferation of autoreactive effector immune cells, therefore helping to protect the body against autoimmunity (25–27). Similarly, RLN2, known as a pregnancy hormone, has been linked with health protective effects including anti-inflammatory actions (28, 29). However, WFA-induced mechanistic regulation of RLN2 and TNFRSF8/CD30 remains to be characterized.

*In silico* analysis revealed that the promoter regions of IL-1β, GM-CSF, CCL2/MCP-1, BAFF, SHBG, and ACRP30/adiponectin possessed NF-κB transcription factor sites; therefore, the collective downregulation of these cytokines/chemokines may be associated with NF-κB regulation. Furthermore, there are no NF-κB binding sites on the CCL17/TARC and TSP-1 promoters. However, transcription factor c-Rel (agtggccaTTCCtaa for CCL17/TARC, and cctggggaTTCCtcc for TSP-1) co-cites with NF-κB anchored at positions 1,061 (+) and 991 (−), respectively, supporting the regulation of CCL17/TARC and TSP-1 *via* NF-κB activation (30–32). Our observation is further supported by a study in smooth muscle cells describing simultaneous induction of multiple cytokines including GM-CSF, CCL2/MCP-1, and PTX3 following IL-1β stimulation (33). Therefore, a collective decrease in multiple cytokines/chemokines may be directly or indirectly associated with the inhibition of a common target such as NF-κB following WFA treatment. Previous studies have also shown that WFA primarily regulates the transcription factor NF-κB signaling pathway by directly binding to the inhibitor of nuclear factor-kappa B kinase subunit beta (IKKβ), resulting in regulation of the diversity of cytokines (34).

To understand the regulation of inflammatory responses, recent emphasis has been placed on investigating the role of inflammasome complex proteins. IL-1β and IL-18 are the best known prime targets of inflammasome activation (35). It is known that cleavage and maturation of IL-1β and IL-18 cytokines are regulated by caspase-1, which is an active component of the inflammasome complex (36). Despite the fact that we did not observe any significant change at the protein level of inflammasome complex proteins (NLRP3: *P* = 0.712, ASC: *P* = 0.319, and pro-caspase-1: *P* = 0.99) following WFA treatment, we see a significant decrease in the production of IL-1β (*P* < 0.0001) and IL-18 (*P* < 0.0001) cytokines. The reduced level of IL-1β secretion may be due to the significant decrease in IL-1β pro-form (*P* = 0.002) following WFA treatment. On the other hand, the protein expression level of IL-18 pro-form is not affected by WFA (*P* = 0.99). However, secretion of IL-18 is significantly decreased (*P* < 0.0001), which could be due to inhibition of active caspase-1 (*P* < 0.0001) following WFA treatment. It has previously been shown that localization of ASC in the cytosol is essential to make a complex with pro-caspae-1, thereby activating inflammasome complex (37). Therefore, it is likely that alterations in the distribution and fragmented localization of NLRP3 and ASC proteins following WFA treatment, without affecting the protein levels of NLRP3, ASC, and pro-caspase-1, inhibits the transformation of pro-caspase-1 to active caspase-1. Although there are other pathways resulting in IL-1β and IL-18 secretion (38), our study suggests that in addition to NF-κB, inflammasome complex could be a new target of WFA, as shown by the mosaic distribution of NLRP3 and ASC proteins, and reduced level of mature IL-18 secretion.

In summary, this study highlights the mechanism by which dietary agent WFA modulates multiple cytokines and chemokines associated with inflammatory diseases *via* inhibition of NF-κB activity and fragmented localization of the NLRP3 inflammasome complex proteins. While IL-1β and IL-18 are the prime targets of inflammasome activation, our observations indicate that the cytokines/chemokines found to be downregulated following WFA treatment could also be potential targets in the signaling cascade of inflammasome activation and need further investigation. Since inflammasomes are linked with multiple autoimmune and auto-inflammatory diseases (39), the use of dietary agent WFA provides a unique opportunity to explore the mechanistic regulation of inflammatory cytokines/chemokines in multiple disease types associated with inflammation.

## Author Contributions

SD, HY, and DK performed the experiments. SD contributed to the writing. DK designed the experimental approach, analyzed the data, and wrote the manuscript. MC, MN, and PN contributed to the discussions. All the authors read and approved the final manuscript.

## Conflict of Interest Statement

The authors have no financial conflict with the subject matter or materials discussed in the manuscript.

## Supplementary Material

Figure S1 Effects of withaferin A (WFA) on proteins of the NLRP3 inflammasome complex and its downstream targets following monosodium urate (MSU) stimulation of THP-1 cells. WFA inhibits the production of caspase-1, interleukin-1 beta (IL-1β), and IL-18 in a dose-dependent manner. Lipopolysaccharide (LPS)-primed THP-1 cells (5 × 10^5^ cells) were stimulated with MSU (200 µg/mL) for 30 min. Following incubation, different doses of WFA (5, 10, 20 µM) were added for 4 h. Cellular proteins were harvested and assayed for the protein levels of NLRP3, pro-caspase-1, IL-1β, and IL-18 by western blot analysis, while the production of active caspase-1, IL-1β, and IL-18 in culture supernatant was determined by enzyme-linked immunosorbent assay (ELISA). Statistical comparisons were performed using one-way analysis of variance, and the significant levels are shown as compared with LPS treatment (control; ns, non-significant). The experiment was repeated twice from independent cell preparations with similar results.

Figure S2 Representative photograph of confocal fluorescence images (magnification 40×) of THP-1 cells analyzing the effect of dietary agent withaferin A (WFA) following NLRP3 inflammasome activation using adenosine triphosphate (ATP). ASC and NLRP3 proteins were visualized by using Cyanine Cy^TM^3 (red-fluorescent) and Alexa Fluor 488 (green-fluorescent) dye labeled antibodies, respectively. DAPI was used for nuclear staining. A merged image of ASC and NLRP3 proteins shows co-localization of inflammasome complex proteins (yellow; marked by arrow). The experiment was repeated twice from independent cell preparations.
