# Supplementary figures and images for: Withaferin A Associated Differential Regulation of Inflammatory Cytokines
Source: Front Immunol. 2018 Feb 9;9:195. doi: 10.3389/fimmu.2018.00195 (PMC5811468; doi:10.3389/fimmu.2018.00195)

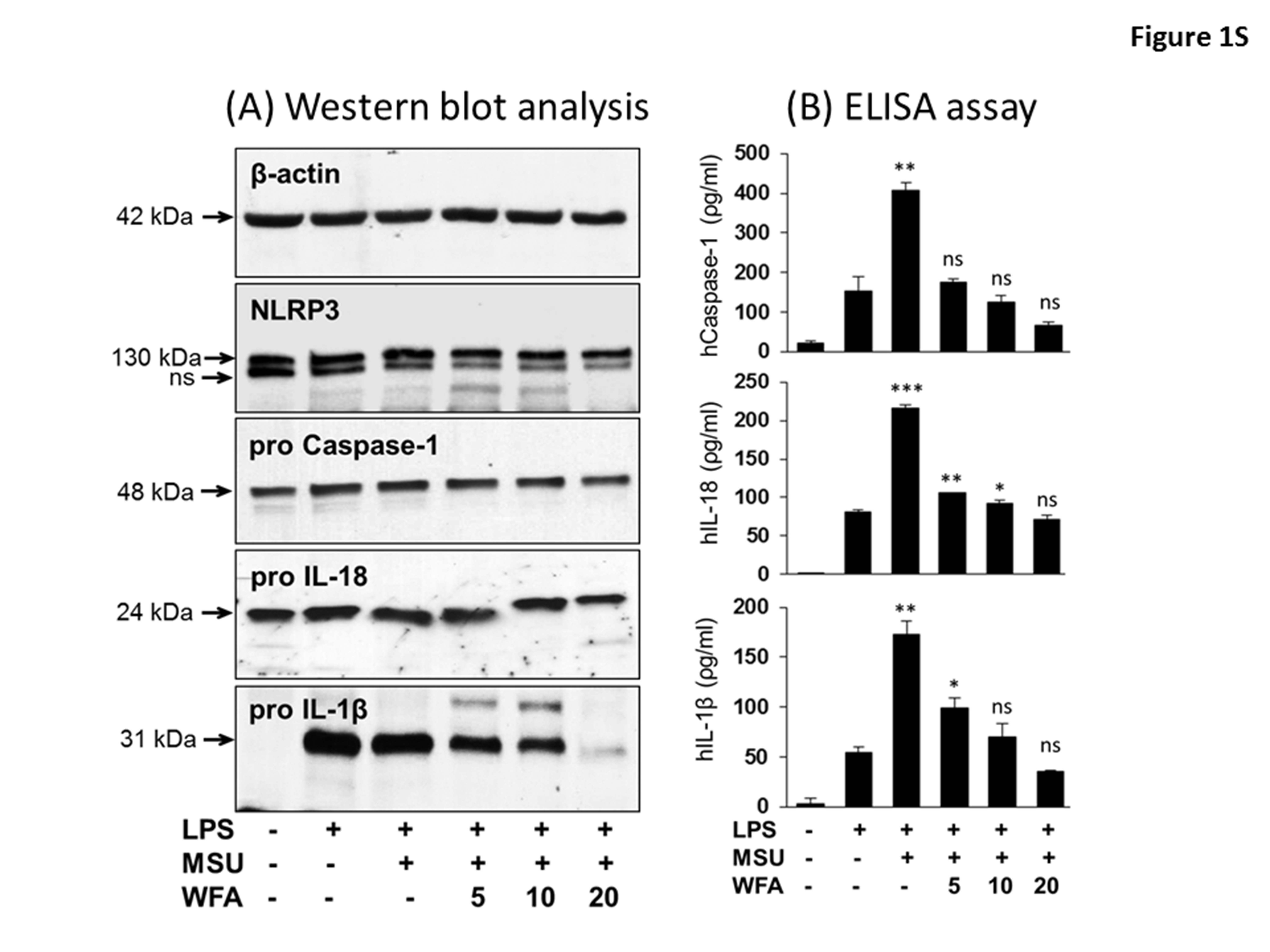

Supplement: Figure S1 — Effects of withaferin A (WFA) on proteins of the NLRP3 inflammasome complex and its downstream targets following monosodium urate (MSU) stimulation of THP-1 cells. WFA inhibits the production of caspase-1, interleukin-1 beta (IL-1β), and IL-18 in a dose-dependent manner. Lipopolysaccharide (LPS)-primed THP-1 cells (5 × 10^5^ cells) were stimulated with MSU (200 µg/mL) for 30 min. Following incubation, different doses of WFA (5, 10, 20 µM) were added for 4 h. Cellular proteins were harvested and assayed for the protein levels of NLRP3, pro-caspase-1, IL-1β, and IL-18 by western blot analysis, while the production of active caspase-1, IL-1β, and IL-18 in culture supernatant was determined by enzyme-linked immunosorbent assay (ELISA). Statistical comparisons were performed using one-way analysis of variance, and the significant levels are shown as compared with LPS treatment (control; ns, non-significant). The experiment was repeated twice from independent cell preparations with similar results. [file image_1.tif]

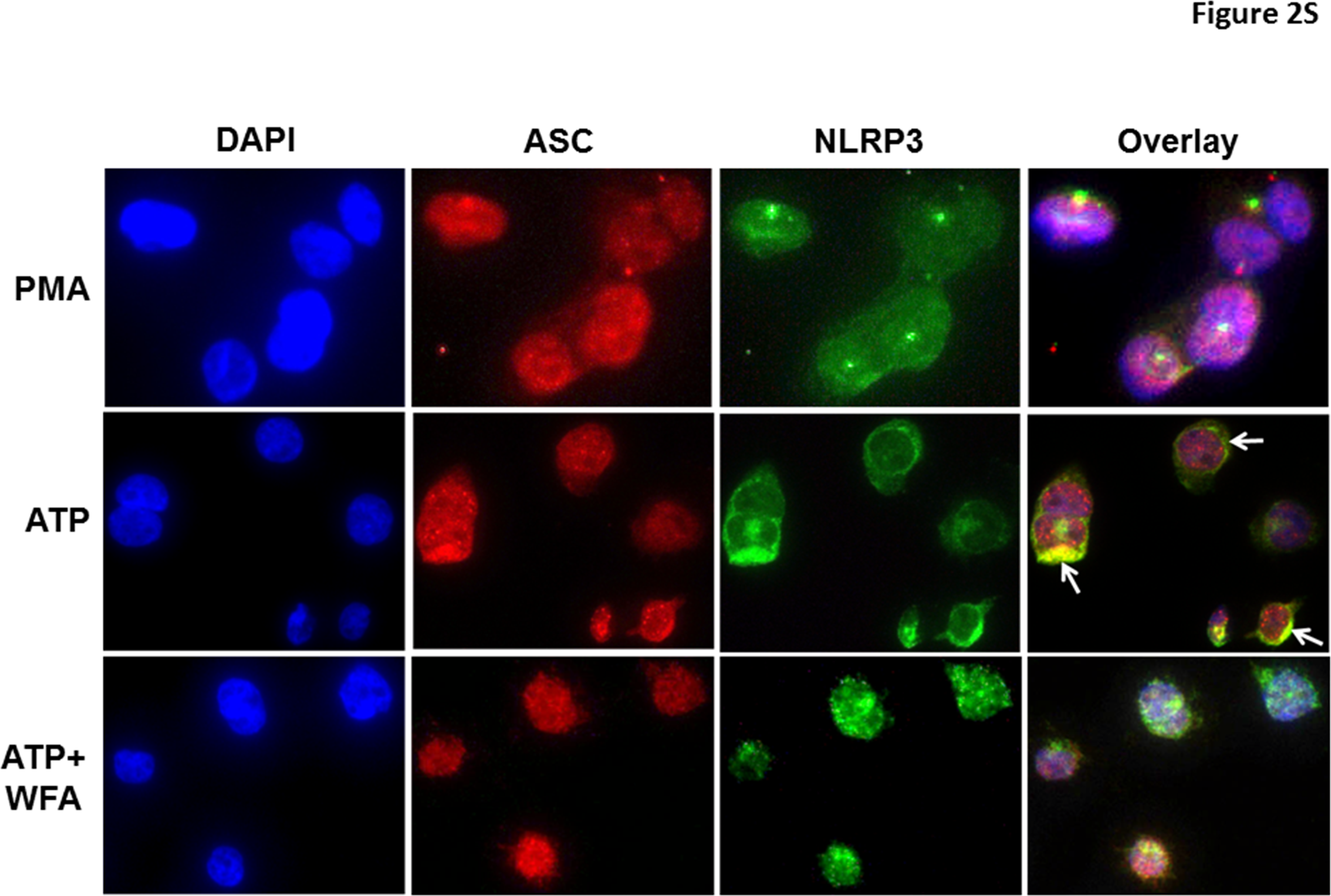

Supplement: Figure S2 — Representative photograph of confocal fluorescence images (magnification 40×) of THP-1 cells analyzing the effect of dietary agent withaferin A (WFA) following NLRP3 inflammasome activation using adenosine triphosphate (ATP). ASC and NLRP3 proteins were visualized by using Cyanine CyTM3 (red-fluorescent) and Alexa Fluor 488 (green-fluorescent) dye labeled antibodies, respectively. DAPI was used for nuclear staining. A merged image of ASC and NLRP3 proteins shows co-localization of inflammasome complex proteins (yellow; marked by arrow). The experiment was repeated twice from independent cell preparations. [file image_2.tif]
