# Supplementary material for: Withaferin A Associated Differential Regulation of Inflammatory Cytokines
Source: Front Immunol. 2018 Feb 9;9:195. doi: 10.3389/fimmu.2018.00195 (PMC5811468; doi:10.3389/fimmu.2018.00195)
Supplement: Supplementary file 3 [file table_1.docx]

**Supplementary Table 1.** List of primers (F: forward; R: reverse) with their reference gene sequence used in Real-Time PCR analysis.

| **Gene** | **Reference sequence** | **Primer sequence (5' to 3')** |
| --- | --- | --- |
| **IL-1β** | NM_000576.2 | F: 5'-AAGTACCTGAGCTCGCCAGTGAAA-3’ |
|  |  | R: 5'-TGAAGGGAAAGAAGGTGCTCAGGT-3’ |
| **CCL2/MCP-1** | NM_002982.3 | F: 5'-GCTCATAGCGCCCACCTTCA-3' |
|  |  | R: 5'-TTCTTTGGGACACTTGCTGC-3' |
| **GM-CSF** | M 11220.1 | F: 5'-ATGTTTGACCTCCAGGAGCC-3' |
|  |  | R: 5'-TTGTAGTGGCTGGCCATCAT-3' |
| **PDGF-AA** | NM_002607.5 | F: 5'-GGATACCTCGCCCATGTTCT-3' |
|  |  | R: 5'-CATGCTTAGTGGCATGGACC-3' |
| **PTX3** | X 63053.1 | F: 5'-TTTTAGTGCCTGCATTTGGG-3' |
|  |  | R: 5'-TCTCCACCCACCACAAACAC-3' |
| **CST3** | NM_001288614.1 | F: 5'-CACGTGTACCAAGACCCAGC-3' |
|  |  | R: 5'-GGCACAGCGTAGATCTGGAA-3' |
| **RLN2** | NM_13441.2 | F: 5'-CAATTTCCAGAGCAGTCGC-3' |
|  |  | R: 5'-GAGGAGCATCTTCCTGGCTC-3' |
| **TNFRSF8 (CD30)** | NM_001243.4 | F: 5'-TTGTGGAGAAGACGCCATGT-3' |
|  |  | R: 5'-ATATCCTGGGGCTTGGTGAC-3' |
| **ACRP30** | NM_001177800 | F: 5'-AGGGAGACATCGGTGAAACC-3' |
|  |  | R: 5'-TGGGCATGTTGGGGATAGTA-3' |
| **GAPDH** | NM_002046 | F: 5'- ACCACAGTCCATGCCATCAC-3' |
|  |  | R: 5'- TCTAGACGGCAGGTCAGGTC-3' |
